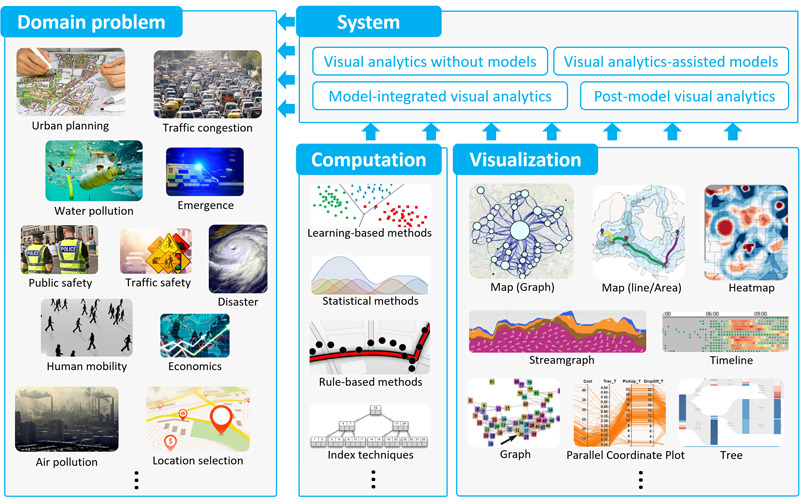# A survey of urban visual analytics: Advances and future directions

**DOI:** 10.1007/s41095-022-0275-7

**Published:** 2022-10-18

**Authors:** Zikun Deng, Di Weng, Shuhan Liu, Yuan Tian, Mingliang Xu, Yingcai Wu

**Affiliations:** 1 State Key Lab of CAD & CG, Zhejiang University, Hangzhou, 310058 China; 2 Microsoft Research Asia, Beijing, 100080 China; 3 School of Information Engineering, Zhengzhou University, Zhengzhou, China; 4 Henan Institute of Advanced Technology, Zhengzhou University, Zhengzhou, 450001 China

**Keywords:** visual analytics, smart city, spatiotemporal data analysis, urban analytics

## Abstract

Developing effective visual analytics systems demands care in characterization of domain problems and integration of visualization techniques and computational models. Urban visual analytics has already achieved remarkable success in tackling urban problems and providing fundamental services for smart cities. To promote further academic research and assist the development of industrial urban analytics systems, we comprehensively review urban visual analytics studies from four perspectives. In particular, we identify 8 urban domains and 22 types of popular visualization, analyze 7 types of computational method, and categorize existing systems into 4 types based on their integration of visualization techniques and computational models. We conclude with potential research directions and opportunities.

### 1 Introduction

Urban computing has achieved remarkable success in tackling many urban problems [1], such as traffic prediction [2], air quality forecasting [3], bike lane planning [4], transit route planning [5], and location selection [6]. However, since urban analytics is an interdisciplinary field, it is crucial to integrate domain knowledge and expertise into the analysis loop. Thus, urban visual analytics [7] is used to empower urban experts using a combination of intuitive data visualization and fast computational methods, enabling experts to visually and interactively perceive, explore, manipulate, and reason about urban data [8].

When developing an urban visual analytics approach, practitioners like urban analysts and researchers may have the following four questions:

1. Which urban *domain problems* have been solved or remain unsolved by visual analytics?
2. What *visualization* techniques have been applied to visually interpret urban data?
3. What *computational methods* have been employed in urban visual analytics to solve urban problems?
4. How are visualization and computational models combined in existing *visual analytics systems*?

Without answers to these questions, researchers will find it difficult to obtain the big picture of the state-of-the-art in urban visual analytics. The lack of such a big picture also prevents urban analysts from quickly finding feasible approaches from prior studies when designing visual analytics solutions for urban problems.

The recent rapid development of urban visual analytics has resulted in an urgent demand for a comprehensive review of how urban problems can be effectively addressed by visual analytics, and to suggest future directions in urban visual analytics [7, 9, 10]. As for question 1, Chen et al.'s [9] and Andrienko et al.'s [10] surveys focused only on the traffic domain. Zheng et al.'s survey [7] discussed general urban visual analytics approaches but was only from a data point of view and did not summarize the domain problems. As for question 2, Chen et al.'s [9] and Zheng et al.'s [7] surveys categorized visualizations based on whether their nature is temporal, spatial, or concerns other

1 State Key Lab of CAD & CG, Zhejiang University, Hangzhou 310058, China. E-mail: Z. Deng, zikun_rain@zju.edu.cn; S. Liu, shliu@zju.edu.cn; Y. Tian, ytian@zju.edu.cn; Y. Wu, ycwu@zju.edu.cn (✉).
2 Microsoft Research Asia, Beijing 100080, China. E-mail: diweng@microsoft.com (✉).
3 School of Information Engineering, Zhengzhou University, Zhengzhou, China. E-mail: iexumingliang@zzu.edu.cn.
4 Henan Institute of Advanced Technology, Zhengzhou University, Zhengzhou 450001, China.
Manuscript received: 2021-12-10; accepted: 2022-02-08

properties. However, many recent techniques are absent from these reviews, as the latest review was published five years ago. As for question 3, no existing survey addresses it. As for question 4, Zheng et al. [7] classified systems that combine visualizations and computational models as "data exploration and pattern interpretation" or "visual learning" according to the system's output. However, how to combine visualizations and computational models, and their respective roles and interrelationships, remain unclear.

In this study, we attempt to develop a systematic and comprehensive survey of urban visual analytics. By answering the four questions, this survey summarizes urban visual analytics studies from multiple perspectives, including domain problem, visualization, computational method, and system. Since urban visual analytics is an interdisciplinary research topic, we investigated prominent journals and conferences in multiple fields, including visualization, transportation, data mining, and geography. To avoid missing important papers, we generally followed the reference- and search-driven paper collection methods used in Guo et al.'s study [11]. The collected papers were mainly published in four journals: IEEE TVCG, CGF, IEEE TITS, and ACM TIST, and four conferences: IEEE VIS, EuroVis, PacificVis, and ACM CHI, between 2007 and 2022. An interactive tool for exploring these papers is available at `https://urban-va-survey.github.io/`.

Our contributions can be summarized as follows:

- *Survey.* We present a systematic survey of urban visual analytics, comprehensively summarizing the significant progress urban visual analytics made in the past few years; four perspectives described below are considered. This survey also indicates future research directions and opportunities.
- *Domain problem.* We categorize the problems studied in urban visual analytics into 8 domains. This not only assists urban analysts in finding relevant visual analytics approaches for real-world urban problems, but also reveals gaps between existing approaches and urban problems that are yet to be solved.
- *Visualization.* We categorize the urban data visualization techniques for spatial, temporal, and other properties into 22 types and demonstrate their use in different analytical tasks, in the hope of promoting better usage and design of urban data visualization.
- *Computation.* We categorize the computational methods that drive urban visual analytics into 7 types and report the usage of artificial intelligence.
- *System.* We categorize 4 types of urban visual analytics system based on the approach to integration of visualization techniques and computational models, and discuss application scenarios and trends for each system type.

### 2 Domain problems

Existing surveys [7, 9, 10] have focused on data and attempted to answer what could be done with different types of data. However, real urban analytics scenarios generally start with specific urban problems. In this section, we classify the urban domains that have been studied by visual analytics into 8 categories: traffic, environment, business, economics, public security, architecture, public service, and public opinion. Tables 1 and 2 summarize the categories, specific problems, and relevant urban visual analytics studies.

Our classification is based on the papers' introduction, case studies, and usage scenarios. Note that some approaches can be applied to multiple domains and address multiple specific problems. Taking VisCas [65] as an example, urban analysts can use VisCas to analyze cascades of both air pollution and traffic congestion events. Hence, we place VisCas into the environment and traffic domains based on applications indicated in its usage scenarios.

#### 2.1 Traffic

The traffic domain is the most widely studied due to the explosive growth of mobility data in the past decade. Mobility data contain valuable knowledge because mobility is the most important manifestation of citizens' activities in urban space.

##### *2.1.1 Human mobility*

Some approaches have been proposed specifically for studying human mobility. They are not limited to a specific problem but provide information in a wide range of scenarios.

Mobility data collected by GPS are usually inaccurate owing to measurement errors and the low sampling rate. Map-matching [81, 82] is the

**Table 1** Domain problems studied by existing urban visual analytics approaches (continued in Table 2)

| Domain | Problem | | Papers |
|---|---|---|---|
| Traffic | Human mobility | Errors and uncertainty of trajectories | [12–15] |
| | | Mobility patterns | [14, 16–31] |
| | | Origin–destination patterns | [25, 32–40] |
| | | Human co-occurrence | [13, 41–43] |
| | | Mobility semantics | [44–47] |
| | Road network | Road network accessibility | [48–50] |
| | | Road centrality | [51] |
| | | Road correlation and causality | [52, 53] |
| | | Intersection interchange behavior | [54–56] |
| | | Tidal lanes analysis | [56, 57] |
| | | Traffic situation understanding | [22, 56] |
| | | Traffic zone division | [19, 58, 59] |
| | Congestion | Congestion monitoring | [60] |
| | | Congestion discovery | [16, 29, 61–63] |
| | | Congestion propagation | [64] |
| | | Congestion events' cascades | [65] |
| | | Congestion causal inference | [29, 56, 61] |
| | | Congestion prediction | [60] |
| | Public transportation | Network accessibility | [66] |
| | | Bus schedule analysis | [67] |
| | | Interchange behavior | [55] |
| | | System usage | [35] |
| | | System efficiency | [68–70] |
| | | Bus network optimization | [48, 69, 70] |
| | | Shuttle bus planning | [47, 71] |
| | Traffic safety | Tunnel surveillance | [72] |
| | | Vehicle monitoring | [73] |
| | | Abnormal driver detection | [47, 74] |
| | | Traffic violation identification | [54] |
| | | Abnormal traffic pattern detection | [73] |
| | Autonomous driving | Traffic light detection evaluation | [75] |
| | | Semantic segmentation evaluation | [76] |
| | | Autonomous driving action evaluation | [77] |
| | | Autonomous driving system evaluation | [78] |
| | Traffic volume forecasting & simulation | | [79, 80] |

first step toward obtaining clean mobility data. Lu et al. [12] assisted this process with visual analytics. Chen et al. [13] summarized five types of uncertainty in trajectory data and proposed a semi-automatic refinement method. In addition to GPS-based data, Chen et al. [14] worked on the uncertainty of trajectories inferred from sparse geo-tagged posts.

Given clean mobility data, various visual analytics approaches have been developed for studying mobility patterns [14, 19–31], origin–destination (OD) patterns [25, 32–40], co-occurrences [13, 41–43], and mobility semantics [15, 44–47]. These analyses support in-depth understanding of how citizens move within the urban space. Notably, some interesting studies have attempted to infer the semantics of mobility using contextual information such as the surrounding points of interest (POIs) [44, 45]. Such semantics form the basis of convenient text-based mobility queries in Refs. [15, 47].

Other approaches involving human mobility focus on addressing domain-specific problems, and we thus categorize these approaches within other domains.

**Table 2** Domain problems studied by existing urban visual analytics approaches (continued from Table 1)

| Domain | Problem | | Papers |
|---|---|---|---|
| Environment | Air quality | Air pollution situation | [83, 84] |
| | | Air pollution co-occurrence | [85, 86] |
| | | Air pollution propagation simulation | [87] |
| | | Air pollution correlation | [88] |
| | | Air pollution event cascades | [65] |
| | | Air pollution causality | [52] |
| | | Air pollution prediction | [89] |
| | Meteorology | Temperature analysis | [90] |
| | | Correlation of temperature and air pollution | [83] |
| | | Ozone hole boundary changes | [50] |
| | | Climate changes | [91] |
| | | Weather prediction | [92, 93] |
| | Water quality | Water quality understanding | [94] |
| | | Water pollution effect on animals | [95] |
| | Noise | Noise spatiotemporal distribution | [84] |
| | | Correlation of noise and crimes | [96] |
| | Radiation | Nuclear contamination understanding | [97] |
| Business | Location selection | Commercial site selection | [38] |
| | | Billboard selection | [98] |
| | | Store selection | [99] |
| | | House selection | [99, 100] |
| | | Warehouse selection | [101] |
| | | POI selection | [48, 102] |
| Public security | Human-created events | Suspect finding | [47] |
| | | Abnormal event detection | [38, 103, 104] |
| | | Marathon monitoring | [105] |
| | | Crime pattern analysis | [96, 106–108] |
| | | Resource allocation | [32, 109, 110] |
| | | Fire station selection | [48] |
| | | Crisis management | [111] |
| | | Disease analysis | [95, 106, 112, 113] |
| | | Surveillance video inspection | [114] |
| | Natural disaster | Flood impact analysis | [115] |
| | | Evacuation monitoring and understanding | [116] |
| Architecture | Understanding | Human-scale scene sense-making | [117, 118] |
| | | Non-visual city attribute prediction | [119] |
| | | Shadow distribution analysis | [120] |
| | | Location functionality analysis | [121] |
| | | 3D environment exploration | [122, 123] |
| | Planning | Impact analysis of new buildings | [120, 124] |
| | | Urban space design precedent seeking | [125] |
| Economics | Real estate market understanding | | [126] |
| | Spatiotemporal understanding of sales | | [25] |
| | Trade network analysis | | [127] |
| | Economic influence between countries | | [86] |
| Public service | Public service events' hotspot, causality, and emergency | | [128] |
| | Park management | | [23] |
| | Locating new hospitals | | [49] |
| | Lost and found | | [29] |
| Public opinion | Spatial and temporal patterns | | [129–131] |

##### *2.1.2 Road network*

As the foundation of urban traffic, road networks have been analyzed in many visual analytics systems. These analyses can be divided into those that operate at macro-, meso-, and micro-levels.

In macro-level analysis, accessibility globally measures how quickly one place can be accessed from another place in a road network. Accessibility can be computed based on the vehicles running on the roads. To analyze accessibility across the entire road network, density-map-based visualization [49] and a visual query-based approach [48] have been developed. Wu et al. [50] also designed visualizations to reveal how the reachability boundary changes over time. Huang et al. [51] analyzed road centrality (importance) in the road network using the PageRank algorithm.

Meso-level analysis studies relationships between multiple roads. Roads can be grouped into zones based on inherent traffic patterns [19, 58, 59]. Implicit correlations and causality linking roads are visually investigated in Refs. [52, 53].

Micro-level analysis considers individual roads. Wang et al. [56] allowed users to assess roads based on passing trajectories. Zheng et al. [57] and Wang et al. [56] studied bi-directional traffic flows on individual roads, supporting policy-making for tidal lanes. Road intersections can be investigated by visualizing traffic interchange behavior [54–56].

##### *2.1.3 Congestion*

The most notorious road traffic problem is congestion. Detecting and understanding congestion are critical to reducing it. Congestion can be visually discovered based on the velocities of trajectories [16, 29, 61–63]. Lee et al. [60] further developed a visual analytics system for predicting traffic congestion. Congestion events can propagate or spread over space and time, leading to a large area of congestion. Many approaches have been proposed for understanding such processes, shedding light on effective congestion control. Wang et al. [64] were the first to visually analyze congestion propagation in a road network based on its topology. Deng et al. [65] studied the implicit cascading processes of congestion events based on their spatiotemporal relationships instead of road network topology. Tailored visualizations have been designed to support expert inference of the multiple causes of congestion [29, 56, 61].

##### *2.1.4 Public transportation*

Efficient, convenient, and comfortable public transportation can satisfy travel demands, alleviate traffic congestion, and improve cities. Representative public transportation methods include subways, shared bicycles, and buses. Visual analytics can facilitate the evaluation and optimization of public transportation systems.

Evaluation can be based on timetables (or schedules), travel time, and the satisfaction of travel demands. Andrienko et al. [66] exploited planned bus timetables to reveal how a transportation system connected urban space (i.e., accessibility). Palomo et al. [67] compared timetables to the actual service provided to diagnose poor outcomes, e.g., serious delays.

A passenger's trajectory along with interchange behaviors [35, 55] can be inferred from OD data in a public transportation system. Thus, various metrics, such as waiting and transfer time, can be derived from the passengers' ODs and visualized to understand efficiency [68, 70]. Beyond numeric metrics, Weng et al. [69] visually encoded the movements of bus passengers to discover gaps between the current system and travel demands.

Experts can optimize a public transportation system after understanding and evaluating it. For example, Di Lorenzo et al.'s method [70] supports comparisons between old and new routes with respect to derived metrics. Kamw et al.'s method [48] can identify areas unreachable on foot and propose candidate bus stops for them. Liu et al. [71] designed interactive visualizations to allow users to visually evaluate potential bus stations and select ideal ones to create a shuttle bus route. Weng et al.'s method [69] integrates a Monte-Carlo tree search model [5] into a progressive, interactive route replacement procedure.

##### *2.1.5 Traffic safety*

Traffic safety can be improved by traffic monitoring, for which visual analytics can provide an interactive and situation-aware environment. Piringer et al. [72] supported situation awareness in the surveillance of road tunnels. Pu et al. [73] designed a vehicle fingerprint to monitor vehicle movements on roads. Visual analytics can also empower experts to detect abnormal traffic behavior [47, 54, 74]. Timely detection can reduce the occurrence of accidents.

##### 2.1.6 Autonomous driving

Autonomous driving is an emerging research area. Deep learning models are incorporated to understand complex road environments and produce driving actions. Thus, the performance of these models is critical. Recently, researchers have started to use visual analytics to evaluate traffic light detection [75], semantic segmentation [76], action prediction models [77], and even entire systems [78].

##### 2.1.7 Prediction & simulation

Andrienko et al. [80] proposed a visual analytics framework for accessing, forecasting, and developing what-if options. Zeng et al. [79] adopted visual analytics for diagnosing the impact of flow aggregations on traffic prediction. It essentially uses visualizations to improve prediction models based on deep learning. Two approaches were used to predict traffic volumes, but they could be extended to other scenarios, such as predicting traffic speed and travel time.

#### 2.2 Environment

Th environment, on one hand, is of great significance to environmental science and geographic science. On the other hand, it greatly affects people's lives. Widely deployed environmental monitoring stations provide a large volume of environmental data that can be used to gain insights into issues such as water quality, air quality, and meteorology changes. Environmental issues have also been widely studied in the visualization community.

##### 2.2.1 Air quality

Air quality has increasingly been a critical problem, with a profound impact on the economy and health. Qu et al. [83] were the first to propose a visual analysis approach for understanding the air pollution problem. Li et al. [84] proposed a real-time visual query method for retrieving the spatiotemporal distribution of air pollution. To control air pollution, it is important to understand its influence and propagation processes. Various visual analytics techniques have been developed to study its propagation processes based on co-occurrences [85, 86], simulation [87], event cascades [65], correlation [88], and causality [52]. Shen et al. [89] proposed a visual analytics approach combined with deep learning to predict air quality.

##### 2.2.2 Meteorology

Meteorological issues also affect people's daily lives. Gautier et al. [90] overlaid temperature data onto a 3D city model to analyze temperature. Qu et al. [83] designed a representation to reason about the correlation between temperature and air quality. Wu et al. [50] analyzed boundary changes of the ozone hole over Antarctica. Li et al. [91] visualized how climate changes over large geographic areas and long periods of time. Among many meteorological issues, weather forecasts are the most familiar. Weather prediction calibration [93] and comparison of prediction outcomes [92] have been studied using visual analytics.

##### 2.2.3 Water quality, noise, and radiation

Water quality, noise, and radiation are less studied in the visualization community. Maciejewski et al. [95] studied the effects of industrial waste water on animals. Accorsi et al. [94] designed an interface for visually exploring the water quality of rivers. For noise pollution, Li et al.'s method [84] supports fast visual querying of the spatiotemporal distribution of flight noise. Malik et al. [96] explored the spatiotemporal correlation among noise complaints, traffic accidents, and drunkenness. Radiation pollution, especially nuclear pollution, is an extremely harmful environmental problem. Wei et al. [97] allowed the visual understanding of radioactive contamination based on static and mobile sensors.

#### 2.3 Business

At present, the application of urban visual analytics in business intelligence mainly concerns location selection.

##### 2.3.1 Selecting facility locations

Selecting suitable facility locations is important to business profitability. An informed selection process requires integration of an intelligent recommendation model and a human-centered multi-faceted evaluation. Visual analytics is a good tool for that.

Different types of facilities have different selection criteria. Traffic flow needs to be considered in many location selection scenarios, such as commercial sites [38], billboards [98], and stores [99]. Some scenarios have specific criteria. Selecting houses focuses on reachability over time [100]. Selecting warehouses should consider the delivery distance [100]. However, unlike the criteria mentioned above, some aspects cannot be easily quantified, for example, the spatial context. Noting this, Weng et al. [99] developed a context-integrated solution for location selection.

##### 2.3.2 Selecting POIs to visit

In addition to selecting locations for facilities, visual analytics can also help people choose POIs to visit. Li et al. [102] embedded keywords extracted from social media into a metro map, called Metro-Wordle, which allows users to seek, e.g., a restaurant for eating beefsteak, based on keywords within a city. Kamw et al.'s method [48] supports choosing a restaurant whose location is conveniently reachable by friends in different places.

#### 2.4 Public security

Public security aims to protect individuals, property, and objects from threats such as disasters or accidents. Threats can be categorized as human-created events or natural disasters.

##### 2.4.1 Human-created events

Identifying unexpected human-created events as potential threats can be based on various data collected in cities, such as human trajectories [38, 47], social postings [104], and taxi trips [103]. Experts can further investigate any anomalies and take measures accordingly. Organized large-scale activities may also demand emergency responses. For example, marathons should be monitored to ensure that medical assistance is timely [105]. Analyses of this kind should be in near real time, as experts need to quickly find abnormalities and respond to them.

Visual analytics also supports in-depth later analysis for public security, in areas such as crime analysis [96, 106–108], police resource allocation [32, 109, 110], fire station selection [48], crisis management [111], disease analysis [95, 106, 112, 113], and surveillance video inspection [114]. For example, given historic crime data, crime hotspots with frequent occurrences of crime can be visually determined [106–108], and urban experts can take informed action such as appropriate policing.

Traffic safety is also an important part of public security: see Section 2.1.

##### 2.4.2 Natural disasters

Timely responses to natural disasters can reduce many losses. Such decision making scenarios can be enhanced through visual analytics. Huang [115] demonstrated the effectiveness of visual analytics in assessing the impact of floods. Li et al.'s approach [116] allows the visual analysis of the emergency evacuation plan simulation.

#### 2.5 Architecture

Architects and urban planners can use visual analytics to understand urban space and assist in planning.

##### 2.5.1 Understanding

The human-scale environment is the urban space that people are most directly exposed to, e.g., the things people see when walking on the street. Street views are proper materials that help people understand the human-scale environment. Thus, researchers have proposed an efficient query method [117] and an exploration system [118]. These street views can also be exploited to predict non-visual city attributes [119]. In addition to street views, Miranda et al. [120] simulated and visualized shadows in the urban physical environment, which helps understand the environmental quality of public spaces. Zhu et al. [121] explored and analyzed the functionality of locations in urban space.

On a larger scale, a zooming technique by Qu et al. [123] allows users to explore a 3D urban environment in an occlusion-free way. Zeng and Ye [122] visually combined 3D physical entities and numeric urban design metrics to study urban vitality.

##### 2.5.2 Planning

Urban planning requires many considerations. For example, when developing new buildings, their impact should be accessed. Ferreira et al. [124] and Miranda et al. [120] combined information visualization with a 3D urban environment to support such analysis. A representative and mature city can be viewed as a precedent for other developing cities. Miranda et al. [125] visualized human behavior in cities to derive urban space design precedents.

#### 2.6 Economics

Data in economic domains has spatial and temporal characteristics, posing challenges of analysis. Sun et al. [126] analyzed the spatiotemporal development of the real estate market and correlations between multiple economic attributes. Liu et al. [25] extracted and visualized spatiotemporal patterns from sales volumes in different regions. In the era of globalization, the economies of different regions influence each other. Wang et al. [127] analyzed trade networks linking countries. Li et al. [86] used co-occurrence patterns of per capita income data to infer country-wise influences.

#### 2.7 Public services

By public services, we mean those serving society and urban residents. Zhang et al. [128] proposed a visual analytics system for investigating heat, water, gas supply, drainage, and road division issues. Hotspot and causality analyses and emergency discovery were supported in their system. Steptoe et al. [23] visually analyzed tourists' trajectories, and their visiting and communication behaviors in parks, to provide insight into improving park services. Chen et al. [29] connected multi-source heterogeneous urban spatiotemporal data through a novel spatiotemporal visual query, and applied it to finding lost objects. Feng et al.'s method [49] can facilitate locating a new hospital to serve citizens while balancing medical resources.

#### 2.8 Public opinion

Some methods utilize social media data to analyze public opinion in a spatial or urban context [129, 130]. Combined with geographic information, users can have a deeper understanding of public opinion, for example, what opinions the people in a region have. Many studies have studied public opinion in the absence of spatial and urban context [132–139], but in general we consider them to be out of the scope of our survey. Readers can instead refer to Refs. [140, 141].

### 3 Visualizations

In this section, we categorize visualizations used in urban visual analytics studies as spatial, temporal, and other property visualizations. We further specify 22 visualization types in these three categories: see Table 3, differently from previous surveys [7, 9].

#### 3.1 Spatial visualization

As the basis of urban analytics, spatial context is involved in almost all visualization studies. Visual elements are depicted in their spatial contexts, to

**Fig. 1** (A) Examples of dot-on-the-map visualization. (B) Examples of line-on-the-map visualization. (C) Examples of heatmap visualization. Reproduced with permission from Ref. [65] for (A1), © IEEE 2021; Ref. [66] for (A2), © IEEE 2019; Ref. [99] for (A3), © IEEE 2018; Ref. [47] for (B1), © IEEE 2016; Ref. [18] for (B2), © IEEE 2011; Ref. [35] for (B3), © The Authors 2015; Ref. [29] for (C1), © IEEE 2017; Ref. [106] for (C2), © IEEE 2010; Ref. [108] for (C3), © IEEE 2019.

**Table 3** Visualizations in urban visual analytics

| Visualization | | Main usage | Papers |
|---|---|---|---|
| Spatial | Map (dot) | Show distribution | [12, 21, 28, 35, 39, 42–46, 48, 57, 58, 62, 63, 65, 66, 70, 71, 73, 80, 84, 86, 88, 91, 94, 95, 98–100, 102, 104, 110, 111, 117, 118, 126, 142] |
| | Map (line) | Show distribution; visualize movement or influence | [12, 13, 15–21, 23, 28–31, 35, 37, 38, 40, 41, 46–48, 51, 54, 60, 62–64, 74, 77, 80, 98, 101, 102, 107, 114, 143] |
| | Map (heat) | Show distribution | [15, 20, 24, 28, 29, 32, 39, 42, 43, 45, 49, 50, 52, 56, 57, 77, 79, 84, 90, 93, 96, 98, 100, 103, 106, 108–113, 117, 119–122, 125, 127, 131, 142] |
| | Map (glyph) | Summarize or compare multi-dimensional spatial data | [20, 25, 33, 34, 36, 41, 44, 45, 51, 53, 55, 61, 65, 66, 69, 73, 85, 87, 93, 101, 103, 121] |
| | Map (area) | Show regions with the same attributes | [12, 13, 20, 22, 26, 39, 48, 58, 59, 61, 68, 69, 71, 80, 92, 97, 107] |
| | Map (graph) | Visualize movement, influence, or relations | [14, 18, 27, 36, 52, 53, 62, 63, 70, 80, 87, 116] |
| | Flow map | Visualize crowd movement | [22, 32] |
| | 3D map | Provide physical context and a sense of presence | [26, 44, 62, 63, 66, 78, 80, 90, 105, 114–116, 120, 122–124] |
| Temporal | Timeline | Show temporal features | [13–15, 18, 20, 23, 26, 27, 33, 36, 38, 40–43, 45–47, 53, 60–65, 68, 72, 73, 79, 84, 87, 88, 91, 96–98, 100, 103, 105, 107–110, 114, 121, 125, 130] |
| | Line/area chart | Show statistics given two dimensions; show temporal features | [14, 17, 19, 20, 22, 25, 26, 29, 39, 42, 43, 45, 50–52, 57, 61–63, 66, 69, 77, 78, 80, 84, 88, 89, 93, 95–97, 100, 103, 105, 106, 108, 110, 112, 113, 125, 127, 128, 131] |
| | Streamgraph | Show temporal features of multi-objects | [50, 54, 89, 126, 129] |
| | Sankey | Show temporal features of multi-objects | [17, 35, 43, 59] |
| | Others | — | [21, 24, 28, 31, 52, 67, 68, 71, 86, 108] |
| Other property | Bar chart | Show statistics given two dimensions; show temporal features | [14, 15, 17, 18, 20, 21, 23, 25, 28–31, 38–40, 43, 44, 54, 58, 60, 62–64, 67, 69–71, 74–78, 85–87, 90, 93, 96, 98–100, 102, 104, 105, 108, 110, 114, 118, 122, 130, 142] |
| | Tree/graph | Visualize movement, influence, or relations | [24, 25, 29, 42, 51, 64, 68, 83, 87, 94, 116, 127] |
| | Scatterplot | Visualize projected multi-dimensional data; show value distribution given two dimensions | [14, 19, 28, 39, 40, 43, 47, 50, 52, 54, 56, 58, 59, 61, 64, 75, 76, 79, 80, 83, 85, 87, 89, 91, 94, 95, 97, 98, 101, 103, 105, 112, 115, 115, 118, 121, 125, 127, 142] |
| | PCP | Show multi-dimensional data | [19, 21, 28, 31, 42, 47, 54, 57, 58, 61, 75, 78, 83, 85, 118, 122, 124] |
| | Radar | Show multi-dimensional data | [20, 40, 71, 78] |
| | Glyph | Summarize or compare multi-dimensional data | [59, 86, 87, 98] |
| | Matrix | Visualize movement or relations | [31, 40, 42, 58, 69, 76, 91, 99, 121, 127] |
| | Wordle | Show semantics | [14, 19, 102, 104, 111, 129] |
| | Video | Provide details and real-world context | [60, 60, 72, 75–78, 114, 116] |

provide spatial visualizations. Such visualizations enable urban analysis to be performed in a geographic context, e.g., by presenting data distributions and anomalies in geographic space. For more precise insight, we further divide spatial visualizations into eight types.

##### *3.1.1 Map (dot)*

Data points like geographic locations and spatial events can be directly plotted as dots on a map (see Fig. 1(A)). Only simple visual channels, such as size and color, are used (e.g., see Figs. 1(A1) and (A2)). There is even no visual encoding in some cases (e.g., see Fig. 1(A3)), so that a large number of data points can be displayed in a scalable manner without overwhelming information.

##### *3.1.2 Map (line)*

Lines or curves on the map can depict trajectories (like human mobilities) or facilities (like bus and subway routes). For trajectories, line-based visualization intuitively shows mobility patterns. Figure 1(B1) shows the spatial distribution of trajectories involving a target location [47]. Figure 1(B2) shows people's preferences for various routes [18]. For facilities, line-based visualization represents the urban geographic reality that is itself linear [35, 69]: e.g., Singapore subway routes in Fig. 1(B3).

#### 3.1.3 *Map (heat)*

Both continuous and discrete spatial heatmaps are of use. A continuous heatmap is a smooth representation of aggregated geographically located objects, usually generated by kernel density estimation (KDE). Both the lines and dots on the map can be aggregated to generate heatmaps. For example, Liu et al. [98] summarized trajectories' pick-up and drop-off locations in a heatmap in Fig. 9(B). Chen et al. [29] summarized trajectories as heatmaps directly in Fig. 1(C1). The spatial distribution of geographically located events can be modeled using heatmaps: e.g., Maciejewski et al. [106] visualized a syndromic population over space based on syndrome events in Fig. 1(C2).

Discrete heatmaps are called choropleths [108, 144–146]. Semantic geographic space division avoids the use of smoothing algorithms such as KDE. Heat is used to show the values of attributes in each region. For instance, Fig. 1(C3) visualizes the number of crimes in different regions [108].

#### 3.1.4 *Map (glyph)*

Glyphs are effective visualizations for multi-dimensional data [147]. Glyphs on a map can summarize and combine complex data within a spatial context and support comparison and in-depth understanding, thereby serving as an overview to guide further exploration. Geographical information is indicated by glyphs' geographic positions. Visual channels of the glyphs encode temporal information, attributes, or associated information. For example, in Zeng et al.'s glyphs [45], an inner pie chart visualizes the portions of the POIs associated with the location, while the outer radial area chart encodes the weekly temporal distribution of mobility (see Fig. 2(A1)). Cao et al. [103] layered rectangle glyphs on a map to indicate attributes of detected anomalies (see Fig. 2(A2)). Weng et al. [69] derived attributes related to the bus service in various regions and encoded them with radar charts of glyphs (see Fig. 2(A3)).

#### 3.1.5 *Map (area)*

An area (e.g., a polygon) on a map indicates some region with a particular attribute. For example, the area within an isocontour indicates some region with a higher attribute value than a certain level, which is widely used in meteorological visualization (e.g., Quinan and Meyer's method [92] in Fig. 2(B1)). In the transportation domain, areas are used to visualize regions reachable under constraints from a given location [50, 68]. Figure 2(B2) shows a concrete example from Ref. [48]. Voronoi diagrams [148] are also areas on a map. In Andrienko et al.'s study [26] (see Fig. 2(B3)), urban space is divided based on significant locations using a Voronoi diagram. Each region of a polygon is covered by a significant location.

#### 3.1.6 *Map (graph)*

A graph on a map comprises a set of nodes with geographic locations and edges between the nodes. Undirected graphs represent mutual relationships between nodes. For example, in Liu et al.'s work [18] (see Fig. 2(C1)), each edge represents the variety of routes between two locations.

Directed graphs can represent human mobilities (e.g., in Refs. [27, 116]) and spatial influence (e.g., in Refs. [52, 65, 87]). Von Landesberger et al. [27] applied spatial and temporal simplification to massive human mobilities and derived a concise mobility graph; see Fig. 2(C2). Li et al. [116] used the graph in Fig. 2(C3) to visualize how people (or agents) move during an emergency evacuation. Some graph visualizations on a map encode spatial influences [52, 65, 87]. Figure 2(C4) is an example from Compass [52], where edges encode the causal relations between nodes of regions. Using the graphs, urban experts can obtain urban deterioration patterns.

More information can refer to a recent review that comprehensively explores the visualization of geographic networks [149].

#### 3.1.7 *Flow map*

A flow map treats human mobilities as a flow. Such a technique is often used to summarize massive crowd movement on a large spatial scale [22, 32]. For example, Kim et al. [32] visualized citywide movement patterns extracted from non-directional discrete events using flow maps.

#### 3.1.8 *3D map*

3D maps provide a realistic urban context in a more immersive and engaging way than 2D geographic maps [150]. They are commonly used in public security [105, 114–116] and architectural domains [120, 122–124] because they provide a sense of presence. For example, Li et al. [105] and Li et al. [116] adopted 3D views to track people who are moving in the urban space with optimal scene

**Fig. 2** (A) Examples of glyph-on-the-map visualization. (B) Examples of area-on-the-map visualization. (C) Examples of graph-on-the-map visualization. Reproduced with permission from Ref. [45] for (A1), © IEEE 2017; Ref. [103] for (A2), © IEEE 2018; Ref. [69] for (A3), © IEEE 2021; Ref. [92] for (B1), © IEEE 2016; Ref. [48] for (B2), © IEEE 2020; Ref. [26] for (B3), © IEEE 2010; Ref. [18] for (C1), © IEEE 2011; Ref. [27] for (C2), © IEEE 2016; Ref. [116] for (C3), © The Author(s) 2020.

navigation. Ferreira et al. [124] developed a 3D decision making framework, where users can perceive the impact of new buildings on urban space (see Fig. 3(A)).

**Fig. 3** (A) Users can perceive the impact of new buildings on urban space in a 3D environment. (B) 3D urban space is deformed to highlight target buildings. Reproduced with permission from Ref. [124] for (A), © IEEE 2015; Ref. [123] for (B), © IEEE 2009.

Note that the third dimension may result in the occlusion of information visualizations. Qu et al. [123] (see Fig. 3(B)) attempted to alleviate the issues by deforming urban space under constraints [151, 152].

#### 3.2 Temporal visualization

Temporal visualizations display temporal features along a timeline. Such visualizations support time-oriented exploration and analysis, such as identifying the temporal distribution of urban data and trends, as well as drilling down for in-depth reasoning. We classify them into 6 types based on the graphical method.

##### *3.2.1 Timeline*

Timeline-based visualizations refer to visualizations compactly encoded along a timeline. Numerical, Boolean, and categorical data can be visually encoded.

The timeline can be linear or circular. Linear timelines (e.g., in Refs. [53, 61, 65, 100, 103, 125])

are readily accepted by general users. For example, Wang et al. [64] created a horizontal timeline (see Fig. 4(A1)). Each row represents 24 hours of a day and each small grid encodes the (numerical) traffic speed using size and color. Deng et al. [65] designed a folded timeline (see Fig. 4(A2)) to show (Boolean) occurrences of event cascading processes. Each vertical bar indicates that a cascading process occurred during that time period. Andrienko et al. [36] adopted a calendar as the timeline (see Fig. 4(A3)), where (categorical) clustering results were encoded by color.

Circular timelines are more artistic (e.g., in Refs. [14, 38, 40, 87, 121]). They are also easy to understand, as they borrow the clock metaphor. For example, Chen et al. [14] designed a wheel to show the periodic temporal distribution of different mobilities (see Fig. 4(A4)). Similarly, in Deng et al.'s glyph [87], the circular timeline shows temporal occurrences of an air pollution propagation pattern.

#### *3.2.2 Line chart and area chart*

Line charts and area charts use lines or areas above a timeline to show temporal evolution. Like a basic chart, they are widely and undoubtedly accepted by general or expert users and therefore can be safely used in many scenarios (e.g., in Refs. [52, 108, 113, 127]).

A line or area chart combined with a circular timeline can also produce more expressive and artistic visualizations [42, 43, 45]. For example, Wu et al. [42] designed a glyph with a circular area chart to show the temporal distribution of mobilities (see Fig. 4(B)). Zeng et al. [45] wrapped their area charts of in and out volumes around pie charts to design an effective glyph (see Fig. 2(A1)).

Line charts can also visualize non-temporal data, if the $x$-axis does not represent time (e.g., in Refs. [63, 80]).

#### *3.2.3 Streamgraph*

Stacked area charts constitute a streamgraph. Each stacked area is called a flow. Besides the quantity of each variable, a streamgraph also displays each

**Fig. 4** Examples of (A) timeline visualization. (B) Circular area chart. (C) Streamgraph. (D) Sankey diagram. (E) Marey graph equipped with KDE. Reproduced with permission from Ref. [64] for (A1), © IEEE 2013; Ref. [65] for (A2), © IEEE 2022; Ref. [36] for (A3), © IEEE 2017; Ref. [14] for (A4), © IEEE 2016; Ref. [42] for (B), © IEEE 2016; Ref. [54] for (C), © IEEE 2011; Ref. [35] for (D), © The Authors 2015; Ref. [67] for (E), © IEEE 2016.

variable's proportion with respect to the sum of all variables. Many researchers use streamgraphs because of this advantage [50, 54, 126, 129]. Figure 4(C) shows a streamgraph from Guo et al.'s work [54], which visualizes the evolution of multiple traffic flows.

##### *3.2.4 Sankey diagram*

Unlike streamgraphs, Sankey diagrams can show not only the evolution of object groups but also transitions between groups over time [17, 35, 43]. The transitions are encoded by splitting and merging of flows. For instance, the Sankey diagram in Zeng et al.'s work [35] visualizes the paths of passengers coming to a station and their corresponding volumes over time (see Fig. 4(D)).

##### *3.2.5 Others*

Other interesting urban temporal visualizations [21, 24, 28, 31, 52, 67, 68, 71, 86, 108] cannot be classified into the above categories. For example, in Fig. 6(A), journeys starting from a station (leftmost red node) are organized according to the travel time in a parallel isotime fashion: travel time from the leftmost station to any station with the same vertical position are the same. A Marey graph smoothed by kernel density estimation (see Fig. 4(E)) was designed by Palomo et al. [67] to visualize the movements or schedules of buses departing at different time. Such designs were generally proposed for specific data and problems.

#### 3.3 Visualization of other properties

In addition to spatial and temporal information, urban data may also contain high-dimensional, relational, and semantic information, etc. Such data also need to be presented and comprehended visually so that analysts can comprehensively analyze more aspects of urban problems. We identify nine popular visualization components for non-temporal and non-spatial data.

##### *3.3.1 Bar chart*

The bar chart is another basic chart. It can not only show temporal information [85, 108, 130] but also statistical distributions, as its coordinate axis can be discrete [15, 25, 98]. For temporal data, the usage of bar charts is similar to a line (or area) chart, which we do not repeat here. For statistics, bar charts can be used to display and compare attribute values of different items (e.g., outcomes of different predictions).

Bars for multiple attributes can be stacked to give a stacked bar chart. Interesting forms of the stacked bar chart are the ValueChart [153] and LineUp [154] (see Figs. 5 and 9(F)) for use in decision making and ranking scenarios [42, 58, 69, 98–100, 118, 155]; they support intuitive comparison of data points with multiple attributes. By stacking the bars of attributes, the stacked bars' heights encode the sum of the corresponding attributes. In LineUp, users are allowed to interactively choose which attributes and bars to stack, thereby enabling informed decision making and ranking.

##### *3.3.2 Tree and graph*

The nodes in a tree or graph may correspond to geographic positions or other data.

When nodes correspond to geographic positions, tree and graph visualizations usually have the same functionality as they would have when placed on a map. The positions of nodes may be freed by separating them from the map [94, 127], enabling visualization layouts to be improved or optimized for aesthetics, legibility, or faithfulness. In the transportation domain, researchers tend to use tree visualizations to visualize mobilities involving a target location [24, 42, 68], which corresponds to the root node of the tree. For example, in Fig. 6(A), Zeng et al. [68] employed a tree visualization to organize journeys of a transportation system starting from the leftmost station.

Such visualizations are usually coordinated with other multiple views and thus can be related back to their spatial context. Some approaches copy graph visualizations directly from the map as a separate view, in which free layouts provide clearer appearances for investigation [51, 64, 87, 116]. Figure 6(B) shows a graph visualization by Huang et al. [51]. Each edge indicates a link between traffic regions.

If nodes lack geographic positions, they usually represent relationships between process statuses or

Fig. 5 LineUp. Bars for the first two attributes are stacked by users. Reproduced with permission from Ref. [100], © ACM 2018.

**Fig. 6** (A) In a public transportation system, journeys starting from a certain station are organized into a tree, with timeline from left to right. (B) A graph visualization shows traffic links between different regions. Reproduced with permission from Ref. [68] for (A), © IEEE 2014; Ref. [51] for (B), © IEEE 2016.

**Fig. 7** (A) Examples of scatterplots. (B) An example of PCP. Reproduced with permission from Ref. [56] for (A1), © IEEE 2014; Ref. [40] for (A2), © IEEE 2019; Ref. [47] for (B), © IEEE 2017.

data attributes. For example, tree visualizations are used to guide processes of steerable data partitioning in Refs. [25, 88]: the interface of TPFlow is shown in Fig. 11. In Qu et al.'s method [83], graph representations are adopted to present correlations between different air pollutants.

Various studies transform graph data into other forms, e.g., tabular [52, 65]; we do not discuss them here.

#### *3.3.3 Scatterplot*

The scatterplot is another basic chart which can unveil interesting patterns, such as clusters, outliers, trends, and correlations [156].

In a traditional scatterplot, the $x$- and $y$-axes denote semantic dimensions [157], for example, driving velocities and distances of vehicles [56] (see Fig. 7(A1)). Similarities and correlations between the dimensions can be identified. Some scatterplots are generated by reducing the dimensionality of high-dimensional or embedded data, e.g., in Ref. [40] (see Fig. 7(A2)). In such cases the $x$- and $y$-axes have no specific semantics, and are only used to show clusters and outliers. Due to their capability of summarizing high-dimensional data, such visualizations are increasingly popular in urban analytics encountering massive data.

Some approaches replace the projected dots by glyphs encoding the original dimensions, e.g., in Ref. [98] (see Fig. 9(D)). In this way, scatterplots become more informative. Note that using glyphs in a scatterplot may cause occlusion and clutter, so this approach is most suitable when the amount of data is small.

#### *3.3.4 Parallel coordinate plot*

High-dimensional attributes of urban data are common. They sometimes are neither spatial nor temporal, but are important in urban analysis.

The parallel coordinate plot (PCP) is the most widely used high-dimensional visualization method besides the projection-based scatterplot in urban visual analytics. For example, multiple non-geographical attributes of trajectories may be visualized by PCPs [19, 21, 31, 47, 54, 57, 58]. Figure 7(B) shows an example from Ref. [47]. Attributes that describe physical urban environments quantitatively are encoded by PCPs in Refs. [122, 124]. PCPs, together with the 3D Map, support both quantitative and qualitative understanding of the urban environments.

PCPs can handle more dimensions in less space than scatterplot matrices. They are also easier to learn and understand than glyphs.

##### *3.3.5 Radar chart, glyph, and scatterplot matrix*

Radar charts, glyphs, and scatterplot matrices are also suitable for high-dimensional data. Radar charts can be seen as a circular PCP based on a radar metaphor but they can handle fewer dimensions than a PCP, and usually do not support user interaction. Nevertheless, due to the universality and their artistic circular shape, radar charts can be seen in many studies [20, 40, 71, 78]. Glyph-based designs are usually placed on the map, as discussed earlier. Sometimes glyphs are put outside the map, e.g., in scatterplots after projection [98] or side by side [86, 87]. Such layouts can better support comparisons. By contrast, scatterplot matrices are less used in urban visual analytics [118], which may be due to their space inefficiency.

##### *3.3.6 Matrix*

Matrix visualizations are a well-arranged representation usually equipped with color encoding. In urban visual analytics, matrix visualizations can visualize pairwise location relationships, in which each row and column represents a location, and each cell encodes the relationship between the row and column. For example, each cell may encode the traffic volume from a source to a destination [58, 69, 158] or the interconnection between row and column locations [42, 121]. Other classic uses of matrix visualizations include showing classification performance (a confusion matrix) [76] and statistics [31, 99].

##### *3.3.7 Wordle*

A wordle or word cloud [159] is usually used in text-based urban visual analytics [14, 19, 102, 104, 111]. It assists in inspecting massive amounts of text information, usually combined with keyword extraction.

##### *3.3.8 Video*

Video is not essentially a visualization method. Nonetheless, in urban analytics, video is an important way to provide raw information. It is also a very familiar concept. Some researchers will add a video component to their visual analytics approaches, allowing experts to verify the conclusions obtained [60, 72, 75–77, 114].

### 4 Computational analysis method

This section reviews computational data analysis methods used in urban visual analytics, aiming to reveal commonly used methods and their purposes. Practitioners can build on these well-established methods to develop intelligent analysis solutions. Seven categories are identified in this survey. Table 4 summarizes the computational methods.

#### 4.1 Learning-based methods

Learning-based methods learn parameters from the inherent distribution of the given data. These methods do not require much prior knowledge to learn those intrinsic patterns in data that provide useful insights or aid predictions.

##### *4.1.1 Clustering*

Clustering is a basic data analysis operation, which divides data into multiple groups by learning similarities in the data. Data items in the same cluster are similar in some way. Such an operation can reduce the amount of data to be analyzed and provide exploration guidance in visual analysis. As long as a similarity measure is well defined, clustering can flexibly apply to various kinds of data, such as events [112], regions [50], and trajectories [31]. Popular clustering techniques are DBSCAN (e.g., in Refs. [38, 51]) and $k$-means (e.g., in Refs. [50, 112]).

##### *4.1.2 Classification*

Classification refers to labeling data with a given set of categorical tags. In early years, researchers tended to use traditional classification techniques, examples being support vector machines for predicting non-visual city attributes [119], conditional random fields for classifying abnormal trajectories [74], and random-forests for classifying locations as work, home, and others [44]. As powerful neural networks became popular, neural-network-based classifiers became increasingly applied to urban analytics, e.g., for sentiment classification of geo-tagged social media [131], human-scale visual feature identification [118], traffic light detection [75], and autonomous driving decisions [77].

Classification techniques rely on the acquisition and quality of labels. Active learning [44, 74] and semi-supervised learning [61] are efficient mechanisms for obtaining labels. Briefly, model trainers first label a portion of the data. A model trained on it is used to label another portion of data. This process is repeated until the number of training samples meets the requirements.

##### *4.1.3 Representation learning*

Representation learning embeds data into a high-dimensional space by vectorization [161]. In this

**Table 4** Computational methods in urban visual analytics

| Computational method | | Example papers |
|---|---|---|
| Learning-based | Clustering | [14, 17, 18, 22, 23, 27, 28, 31, 34, 36, 38, 42–44, 46, 50, 51, 58, 62, 63, 71, 91, 94, 112, 116, 128] |
| | Classification | [44, 61, 74, 75, 77, 118, 119, 131] |
| | Representation | [40, 75, 76, 87, 117, 121] |
| | Dimensionality reduction | [40, 85–87, 98] |
| | Regression | [52, 80, 119] |
| | Forecasting | [60, 79, 89] |
| Statistical | Kernel density estimation | [32, 38, 40, 49, 50, 67, 84, 96, 106, 107, 109, 110, 112, 125, 131, 142] |
| | Matrix/tensor/time series decomposition | [25, 59, 95, 103, 104, 108, 109, 112] |
| | Deviation-based anomaly detection | [17, 23, 38, 53, 95, 103–106, 127] |
| | Keyword/topic extraction | [14, 15, 19, 102, 104, 130] |
| | Frequent pattern mining | [24, 85–87, 94] |
| | Peak/periodicity/correlation of time series | [26, 52, 88, 96, 127] |
| | Metric/indicator/factors calculation | [18, 19, 21, 30, 51, 57, 61, 78, 83, 101, 131] |
| Rule-based | Association | [15, 19, 41, 42, 45, 46, 49, 85, 86] |
| | Summarization | [24, 27, 36, 57, 70, 72, 79, 80, 97, 106, 112] |
| | Map matching | [12, 17–19, 30, 33, 34, 38, 47, 56, 60, 64, 73, 160] |
| | Traffic modelling | [53, 60, 64, 80] |
| | Data cleaning | [13, 20] |
| | Object tracking | [114] |
| | Heuristic search | [65, 69, 70, 93, 98] |
| Simulation-based | | [16, 32, 87, 113, 120, 124] |
| Mathematical programming | | [105, 116] |
| Indexing | | [15, 17, 22, 24, 30, 33–35, 41, 45, 47, 56, 85, 98, 100, 129, 160] |
| Querying | | [15, 22, 29, 35, 39, 47, 56, 84, 100, 117, 118] |

space, adversarial examples can also be generated for adversarial learning [75, 76]. The vectors capture the inherent relationships between data. Reducing the dimensionality of the data vectors to a low-dimensional space can generate an overview for further exploration.

Representation learning can be based on autoencoder [75, 76] or word2vec [40, 87, 121]; the latter makes full use of the characteristics of spatiotemporal data. For example, in Ref. [121], each location is cast as a word, and each trajectory is cast as a sentence comprising the words it passes through. Thus, the latent semantics of locations are learned like learning the semantics of words.

The above methods often take the input itself or the context of the input as the output, so avoid the labeling process to obtain labeled samples. Some approaches leverage open-source models pre-trained with labels. For example, Miranda et al. [117] input street images into pre-trained image recognition models and obtained the latent vector representations for these images from the hidden layers.

##### *4.1.4 Dimensionality reduction*

Dimensionality reduction transforms ubiquitous multi-dimensional data into low-dimensional data. It is usually combined with 2D scatterplots, which are useful and popular in urban visual analytics [98, 99]. Some high-dimensional embeddings also require dimensionality reduction for visualization, as mentioned earlier. Popular techniques include PCA [75], MDS [85, 98], and t-SNE [40, 86, 87]. Currently, t-SNE is the most effective method, particularly for high-dimensional and large-scale data.

##### *4.1.5 Regression*

Regression quantifies relationships between variables [52, 80, 119]. For example, in Ref. [52], the Granger causality test based on vector autoregression is adopted to detect causal relations between time series. In Ref. [80], a polynomial regression model is applied to capture dependencies between traffic intensity and velocity.

##### *4.1.6 Forecasting*

Forecasting refers to predicting future trends. Understanding development trends is an essential prerequisite for wise urban planning. Time series forecasting based on deep learning has achieved great success, with corresponding visual analytics [60, 79, 89]. Forecasting can be seen as a kind of supervised learning. Fortunately, training samples with labels (i.e., future situations) for forecasting can be constructed using sliding windows in the time dimension, avoiding the labeling process.

#### 4.2 Statistical methods

Statistical methods mainly build on statistics to process data. Because of mathematical theories, such methods are often explainable and can guarantee the reliability of analyses.

##### *4.2.1 Kernel density estimation*

Kernel density estimation (KDE) estimates the probability density function of discrete data to characterize them in a continuous way.

KDE can be directly applied to spatial events [96], locations [110], geo-tagged social media posts [131], trajectories [98, 100], and bus schedules [67], generating clear overviews without clutter. Furthermore, KDE has been extended to many scenarios. Feng et al. [49] proposed a topology density map that considers topological conditions of the road network. Li et al. [84] proposed a peak-based KDE that avoids manually setting the bandwidth.

KDEs are usually equipped with heatmap visualizations, e.g., see Figs. 1(C1) and 1(C2), but other studies use KDEs for purposes other than visualization. Data processed by KDE can facilitate further computation and analysis [106, 109], such as sampling [40], reducing errors [38], prediction [112], flow map extraction [32], and topology feature extraction [107, 125, 142]. For example, Lukasczy et al. [107] used KDE to generate a scalar function for discrete spatial event data which enables subsequent topology analysis.

##### *4.2.2 Matrix, tensor, and time series decomposition*

We place matrix factorization, tensor decomposition, and time series decomposition into this category as they decompose data into multiple components.

Garcıa et al. [108] modeled crime data as a matrix in which each row represents a region and each column represents a time slice. Natrix factorization was then applied to this matrix to extract spatial and temporal patterns represented by decomposed matrices. Tensor decomposition techniques in Refs. [25, 103] mathematically extend matrix factorization. In these two studies, spatiotemporal data are modeled as tensors. Latent spatial and temporal patterns are then extracted by decomposing the tensors.

In contrast, time series decomposition, particularly seasonal-trend decomposition (STL), works on time series data. STL decomposes a time series into multiple series with temporal patterns, such as yearly seasonality, day-of-the-week effect, and global trends [95, 104, 109, 112]. Anomaly detection and prediction can be performed using the decomposed time series.

##### *4.2.3 Deviation-based anomaly detection*

From a temporal perspective, determining whether a data point is abnormal is generally based on how much it statistically deviates from other historical data. To perform the comparison, many methods can be used, such as local outlier factor (LOF) [103], cumulative summation (CUSUM) [95, 106], the minDistort algorithm [53], and extreme value theory [38]. From the sampling perspective, anomaly detection can also be accomplished by measuring the deviation of a data point within the samples [23, 104, 105].

##### *4.2.4 Keyword and topic extraction*

This type of method extracts frequently occurring keywords or implicit topics from document corpora. The corpora can text [14, 102, 104, 130] or textualized trajectories after rule-based association [15, 19] (see Section 4.3.1). For example, Chen et al. [14] extracted frequent keywords for locations from microblogs, enabling semantic analysis of movement data. A different example is Huang et al.'s approach [15], in which each trajectory is assigned the names of the streets it passes through to thereby produce a sentence. This process generates a trajectory corpus, then latent Dirichlet allocation (LDA), a topic modeling technique, extracts the latent topic and related keywords for querying and reasoning about mobility patterns.

##### *4.2.5 Frequent pattern mining*

Frequent pattern mining methods can extract significant patterns with frequent occurrences from various types of urban data, such as transactional [85, 86], sequential [24, 94], and graph [87] data. Frequent patterns can summarize massive data, preventing users from being overwhelmed. For example, Deng et al. [87] adopted frequent subgraph mining to extract the propagation patterns from numerous propagation processes for air pollutants. These patterns are then organized and visualized to analyze air quality deterioration on a large spatiotemporal scale.

##### *4.2.6 Time series analysis*

Time series data are an important data type in urban analysis. Correlation analysis and periodicity identification are common analysis tasks. Correlation analysis is usually based on Pearson's $r$ [52, 96, 127]. Information theory can also be applied [88]. Periodicity can be identified using the Fourier transform [26].

##### *4.2.7 Metric and indicator calculation*

Various indicators can be extracted through statistical methods. Entropy based on information theory is popular because it can process the inherent data distribution to estimate the amount of information [18, 19, 21, 57, 61, 131]. The richer the information, the more worthy it is of analysis. For example, Zheng et al. [57] used entropy to quantify how interesting regions are by incorporating spatiotemporal and mobility-related attributes. Other examples include estimating road importance using the PageRank algorithm in a graph-represented road network [51], and deriving correlation metrics between dimensions in air pollution data [83].

#### 4.3 Rule-based methods

Rule-based methods incorporate effective problem- or domain-related rules to guide data analysis. Like statistical methods, rule-based methods are explainable, but they are preferred by domain experts due to their ability to incorporate domain knowledge.

##### *4.3.1 Association*

Many rule-based approaches can associate different urban data based on co-occurring observations, which enriches data and discloses latent relationships within various urban data.

Associating POIs with mobilities is the most common association operation in urban visual analytics. In Refs. [15, 19, 45, 46], geographic positions of movement records were assigned to a set of POIs spatially near the positions. POIs can also be associated with the road network, enriching semantics to urban facilities for accessibility analysis [49].

Spatiotemporal co-occurrence can also be applied to associating people with other people [41, 42], value ranges with other value ranges [85], and events with other events [86].

##### *4.3.2 Summarization*

Summarization is often necessary in visually analyzing massive urban data.

Andrienko and Andrienko [162] demonstrated that movement summarization is an important step towards scalable and effective urban analyses. In many practical approaches, mobilities are aggregated by origin and destination, and summarized as flows between them [27, 36, 57, 70, 80]. Visiting sequences are aggregated and summarized visually according to their shared parts in Ref. [24].

The aggregation of spatial geo-tagged data [97, 106, 112] and spatial regions [27, 79] at various granularities is also seen in many approaches. These summarization procedures can reduce visual clutter on a map and can protect data privacy.

##### *4.3.3 Map matching*

Map matching is a fundamental process for movement analysis. Position shifts exist in movement data collected by GPS devices. Map matching maps these GPS records onto real road networks: vehicles and people can only move on roads, allowing the generation of clean and usable movement data. Thus, many applications using GPS data adopt map matching techniques (e.g., Refs. [47, 64, 160]).

##### *4.3.4 Heuristic search*

Optimal solutions to some urban analysis problems cannot be obtained directly and quickly. Thus, researchers incorporate rules into heuristic search methods to solve such problems.

Greedy search is one of the most popular heuristic search methods. In billboard selection [98], the billboard locations should cover as many trajectories as possible to have high exposure. Such a problem is essentially a $K$-cover problem [6, 163]. In cascading pattern inference [65], extracting cascading patterns that best describe the observed event data is also a $K$-cover problem [164]. In these scenarios, greedy search methods are used because of the NP-hardness of the $K$-cover problem.

Heuristic search can also be used to improve a bus network. In Di Lorenzo et al.'s AllAboard [70], a public transportation system is improved by minimizing users' travel time under constraints. Di Lorenzo et al. considered it a separable programming problem and adopted a heuristic procedure to approximately solve it. In Weng et al.'s BNVA [69], bus network improvement is seen as a multi-objective optimization problem considering cost, passenger volume, directness, etc. They used a heuristic method, Monte Carlo tree search to solve it. Heuristic strategies have also been used for OD-flow sampling [40], and majority voting for weather forecast calibration [93].

##### *4.3.5 Traffic modeling, cleaning, and tracking*

Domain-specific rules also guide traffic modeling, data cleaning, and tracking.

Vehicles are physically constrained to run on the road and to follow traffic rules. Therefore, traffic congestion propagation can be estimated based on the topology of the road network [60, 64]. Moreover, the road traffic can be simulated based on similar heuristic rules [53, 80]. Various data cleaning operations are also based on rules. Ma et al. [20] defined Ping-Pong effects in mobility data as frequently switching between different locations and proposed detection and elimination algorithms. Chen et al. [13] considered five types of uncertainty in human behavior data and developed a semi-automatic processing framework. Finally, Meghdadi and Irani [114] tracked people from consecutive video frames based on continuity of movement.

#### 4.4 Simulation-based methods

Simulation-based methods simulate real-world conditions based on physical phenomena. They effectively recover the real-world urban situation and analyze its development and evolution.

Ray tracing has been utilized to compute the impact of new buildings in terms of shadows [120] and visibility [124]. A gravity model was utilized by Kim et al. [32] to construct flow fields from discrete event data. A particle advection technique helps to generate and visualize the 2D vector fields of traffic flow in Ref. [16]. These methods have potential for generalization. Domain-specific models based on epidemiology and fluid mechanics have also been employed for epidemic response evaluation [113] and air pollution propagation modelling [87], respectively.

#### 4.5 Mathematical programming

Some analysis problems can be characterized as operation research problems, solved by mathematical programming. Although mathematical programming methods can obtain globally optimal solutions, they are used infrequently, perhaps due to their poor scaling to large amounts of data.

MaraVis [105] computes the optimal camera path for monitoring marathons by solving a traveling salesman problem. SEEVis [116] also computes the optimal camera path but for exploring human movements during an emergency evacuation.

#### 4.6 Indexing and querying

Indexing techniques underpin efficient data retrieval, computation, and visualization.

Many urban visual analytics approaches incorporate well-established spatiotemporal index methods, such as the $B^+$ tree [85], quadtree [17, 30, 41], octree [118], $k$-d tree [39], locality sensitive hashing [117], and space-time cube [29]. In addition to these general indexes, other indexes are tailored for trajectory computation and visualization. Location-trajectory indexes enable efficient trajectory retrieval by locations visited [22, 24, 35, 56, 98, 100, 160]. Trajectories can also be indexed by text, after the locations they visit are textualized by associating POIs [15, 47]. With these indexes, the system can support flexible semantic queries.

With the increasing amount of urban data, many advanced indexing techniques have been proposed in the database community [165, 166]. Being aware of the uniqueness of visualization and visual analytics tasks, visualization researchers have proposed big data indexing and management approaches for visualization purposes [84, 129, 167–171].

### 5 Systems

Due to the spatiotemporal, heterogeneous, uncertain, and dynamic characteristics of urban data, it is often necessary to integrate machines and human intelligence in the analysis process. This section considers how computation and visualization combine in a human-in-the-loop urban analytics system. Unlike a previous survey [7], we focus on the role of computation and visualizations and how they interact in a system. Four categories are identified based on how models and visualizations are combined: visual analytics without models, post-model visual analytics, model-integrated visual analytics, and visual analytics-assisted models.

Note that the models mentioned in this section may comprise multiple computational methods from the last section. For example, although the location selection model of Liu et al. [98] comprises map-matching, indexing, and optimization modules, it is viewed as one model from the perspective of an entire system.

### 5.1 Visual analytics without models

The systems in this category exclude models. They are suitable for those scenarios where the raw data do not need to undergo complex computational transformations. Urban analyses mainly rely on well-designed data visualizations.

#### *5.1.1 Individual visualizations*

For an emerging field and problem, data visualizations alone often contribute sufficiently to a domain [55, 83, 130, 172, 173]. The most obvious evidence is in the study of human mobility visualization. In the early years when mobility data started to be collected, the presentation of mobility data provided sufficient insights [55, 172, 173]. Similar evidences can be observed in air pollution [83] and geo-tagged social media analyses [130].

#### *5.1.2 Coordinated visualizations*

As data becomes increasingly abundant and tasks become increasingly challenging, multiple coordinated visualizations become important [18, 20, 33, 34, 41, 50, 54, 72, 73, 91, 97, 99, 110, 126, 127, 174]. To handle large-scale data, these systems generally follow a workflow based on the information-seeking mantra [175]: "Overview first, zoom and filter, then details-on-demand." For example, VisMate [91] first provides a spatiotemporal summary of climate data collected by all meteorological stations. Users can then zoom into a cluster of stations of interest and finally individual stations. Similarly, Liu et al.'s method [18] allows users to analyze the numerous trajectories going from region, to trip, and finally road.

Completing complex tasks also raises the same requirement of coordinated visualizations, although when data are not so large. For instance, AlVis [72] coordinates multiple visualizations to support tasks of tunnel surveillance, such as navigation, event prioritization, video retrieval, and situation predictions.

### 5.2 Post-model visual analytics

In post-model visual analytics systems, the role of computational models is to discover knowledge from urban data in advance. Once the model has run, it rarely needs to be adjusted and run again. Visualizations are designed to organize and present the discovered knowledge. We further classify these systems based on the specific purposes of the models: pattern extraction, item detection, and data enrichment.

#### *5.2.1 Pattern extraction*

Models first extract many patterns from the original data. Visual analytics systems then provide understanding of these extracted patterns, such as co-occurrences [42, 43, 85], mobility [24, 27, 31, 32, 40], air pollution propagation [87], and social media distribution patterns [102, 104, 129, 131]. The huge number of patterns may hinder identifying and reasoning about valuable ones, so a well-designed pattern organization is desirable. For example, Wu et al. [42] extracted co-occurrence patterns in human mobility from trajectory data and and provides multiple visualizations for pattern exploration at different levels and from different perspectives. Deng et al. [87] extracted many propagation patterns of air pollutants from propagation processes, and use a hierarchical organization for effective top–down exploration (see Fig. 8).

#### *5.2.2 Item detection*

Models can detect important items within a collection of data. In urban scenarios, it is common to detect important locations within a vast geographic space [62, 63], such as those with frequent movement [57], traffic jams [61], places of heightened activity [125], and crime hotspots [106, 107]. Starting from these locations, visualizations help users to understand these locations and what happened there. For instance, in Pi et al.'s traffic congestion analyses [61], roads detected as having potential traffic jams and the inferred causes provided a way into the analysis.

#### *5.2.3 Data enrichment*

In many systems, models enrich the original data by deriving additional information [21, 45, 46, 68, 114, 118, 121], such as transportation system metrics [49, 51, 70, 122], clusters [19, 23, 58, 59, 128], and temporal predictions [112].

Taking transportation network diagnosis for example, accessibility [49] and centrality [51] of road networks are calculated and visualized together with the networks. Human mobilities may be estimated and visualized with public transportation networks

**Fig. 8** Views in AirVis from left to right hierarchically organize numerous propagation patterns of air pollution. Reproduced with permission from Ref. [87], © IEEE 2020.

to diagnose system efficiency [68] (see Fig. 6(A)). The additional information over simple visualization alone is important in understanding transportation systems.

#### 5.3 Model-integrated visual analytics

The utility and intelligence of a system can be significantly improved by tightly integrating computational models with visual analytics. Visualizations and computational models can then interact frequently. Here are some typical scenarios.

##### *5.3.1 Problem-specific analysis*

In many specific problems, users need to determine a subset of data of interest through spatial [30, 65, 66, 69, 94, 98, 101, 108], temporal [12, 71], spatiotemporal [17, 52, 100, 109], or other property visualizations [86]. The subset is fed into the models, and the outputs are visually displayed.

Unlike post-model visual analytics systems, these systems require visualizations to determine the inputs to the model. For instances, billboards should be placed in a solution area and viewed by target audiences from a target area. Thus, SmartAdP [98] first requires users to specify target and solution areas on a map based on a visual trajectory overview (see Fig. 9(B)). Next, candidate billboard locations are extracted, based on the areas and trajectories. The next step is to visually compare and evaluate these candidate locations (see Figs. 9(D)–9(F)). In the study of BNVA [69], users were firstly required to conduct network-level analysis on the map and identify those bus routes with low efficiency. Then, an optimization method manipulated each target bus route, and users visually evaluate the optimized routes.

##### *5.3.2 Multi-step analysis*

If an analysis workflow contains multiple steps, each step may involve models and visualizations. In a study on trajectory data cleaning [13], users iteratively interact with different modules of the model through visualizations to address the uncertainty in different dimensions.

##### *5.3.3 Iterative analysis*

Another type of analysis pipeline contains a loop where models can iteratively learn from users' feedback and be updated accordingly [44, 74, 103]. Taking anomaly detection as an example, Liao et al.'s method [74] adopts active learning to interactively learn from samples labeled by users. Cao et al.'s method [103] applies user feedback through Bayesian theory. In this way, models perform better under the supervision of human knowledge.

##### *5.3.4 Query-based analysis*

Visual queries allow users to repeatedly modify query conditions and issue queries to obtain desired results [15, 22, 35, 39, 47, 48, 56, 84, 117]. For

**Fig. 9** Interface of SmartAdP, a visual analytics system for selecting billboard locations based on taxi trajectories. Reproduced with permission from Ref. [98], © IEEE 2017.

example, Ferreira et al. [39] allowed users to query taxi trips in different geographic areas. In Ref. [56], two queries are issued to identify a low-capacity watershed-like intersection.

*5.3.5 Monitoring*

Urban monitoring models should run continuously [60, 103, 105, 116, 176]. The time-varying state of the city is reflected by the data collected by sensors. To monitor cities in (near) real time, the models should continuously integrate streaming and historical data and output new results, such as predicted traffic congestion [60] and detected anomalies [103].

#### 5.4 Visual analytics-assisted models

Urban visual analytics can be a great aid if models serving cities need to be diagnosed, adjusted, or improved. In this context, improving model performances is the purpose.

Representative examples provide model diagnoses [75–79] and steering [25, 80, 88, 93, 96]. For example, autonomous driving models and systems include artificial-intelligence-based decision-making models that run in the urban space. Diagnosing and improving them demand visual analytics system with knowledge of urban context [75–78]. Figure 10 shows Jamonnak et al.'s system for autonomous driving model diagnosis [77]. In this system, users can assess model performance within a spatial context. Steering spatiotemporal analysis models may require interactive visualizations, such as the voting framework for weather forecast calibration [93], tensor decomposition for spatiotemporal pattern

**Fig. 10** Interface of a geographical-context based diagnosis system for autonomous driving models. Reproduced with permission from Ref. [77], © IEEE 2022.

extraction [25], traffic analysis, and forecasting [80]. For example, Liu et al. [25] designed the tree in Fig. 11

**Fig. 11** Interface of TPFlow, a visual analytics system that supports steering tensor decomposition and analysis of spatiotemporal patterns. Reproduced with permission from Ref. [25], © IEEE 2019.

which can be split and expanded interactively to steer the tensor decomposition. In a model steering process, every step of the model is transparent, controllable, and reliable to analysts. Models improve under the supervision of human knowledge.

## 6 Challenges in urban visual analytics design

This section summarizes four high-level challenges when designing a visual analytics approach and discusses feasible solutions.

### 6.1 Problem characterization

Urban visual analytics is an application-driven field. After domain experts put forward their problems and requirements, visual analytics designers must disassemble and characterize them into problems that can be addressed by appropriate visualization techniques and computational methods. Characterizing the problem well requires close and long-term collaboration with experts [177]. Such a challenge generally exists in the development of other visual analytics methods [178, 179].

### 6.2 High-dimensionality and heterogeneity

Urban data usually involves high-dimensional and heterogeneous attri-butes, such as spatiotemporal data [86], text [129], images [117], and 3D physical models [124], which poses challenges for urban data visualization. Two strategies can solve these challenges.

The first is to coordinate multiple visualizations in a visual analytics system. In this way, different visualizations show different and heterogeneous dimensions of urban data: see, for example, TPFlow [25] in Fig. 11 and StreetVizor [118]. Although this strategy is effective and widely used, designers should keep interaction and context switching costs in mind. The second approach is to design integrated visualizations. Different aspects of urban data are encoded together intuitively, and can be analyzed within the same view and context. For example, Sun et al. [152] embedded temporal data into graphically broadened roads. Glyphs in Fig. 2(A1) visualize spatial, temporal, and categorical attributes [45]. This strategy is more difficult than coordinating visualizations because it needs to leverage limited visual channels appropriately in a confined space.

### 6.3 Scalability

Ubiquitous sensors in urban environments continuously monitor a city, generating massive amounts of raw data. Furthermore, the hidden patterns can sometimes be numerous. To accommodate the sheer volume of such urban data, scalability issues must be addressed from visualization and computation perspectives.

#### *6.3.1 Visualization*

To avoid being overwhelmed, users expect data and visualizations to be well organized. The most famous organization follows the information-seeking mantra [175]: "Overview first, zoom and filter, then details-on-demand." Designing an overview and drilling-down in interactive exploration must consider domain requirements. If an inherent hierarchy exists, the corresponding hierarchical organization is desirable: see, for example, AirVis [87] (see Fig. 8) and BNVA [69]. Such a mechanism for hierarchical exploration can be called a level-of-detail mechanism [85].

In addition, graphics optimization can make visualizations more readable by reducing visual clutter. For example, edge bundling techniques [180] can bundle multiple trajectories [37]. Element ordering can reduce visual wiggles and crossings [135, 181]. Sampling techniques that consider visual perception can reduce visual occlusion [40, 182–187].

#### *6.3.2 Computation*

Large amounts of data also slow down computation and prevent seamless interaction. Accordingly, indexing techniques [84, 129, 167–171], progressive analysis [25, 69, 188], approximation [189], GPU rendering [190, 191], etc., can be incorporated into systems to accelerate the computation. Effective indexes ensure fast access to data. Approximation sacrifices a small amount of accuracy to improve speed. Progressive analysis returns results continuously rather than only providing a final result after a time-consuming computation. Progressive analysis can be adjusted or stopped on the fly. GPU rendering focuses on efficient rendering of graphics elements rather than data computation.

### 6.4 Uncertainty

Urban data can be inherently uncertain due to insufficient spatial and temporal granularity or imprecision and errors at sensor terminals [12, 13, 15].

There are two feasible actions to alleviate uncertainty. The first is to design uncertainty-aware visualizations [145, 192–194] for key steps in data processing and transformation. For example, in cascading pattern inference [65], occurrences of instances that contradict the pattern inference result are visually emphasized to users. In the natural-language-based trajectory query [15], a relevance tree is used to show uncertainty in semantic matching between natural language and POIs. In movement semantics enrichment [46], a gradient colormap is applied to visualize uncertainties when assigning a POI to a destination point of movement.

The other approach is to allow users to inspect the raw data for validation. This action is naive but practical in many systems [52, 65].

### 7 Future directions and opportunities

Although urban visual analytics has made remarkable achievements, there are still gaps to be filled. Ever-changing urban life also raises new requirements for urban visual analytics. This section intends to illustrate gaps and requirements.

#### 7.1 Domain problems

We give two potential domain problems that could be investigated in future; data availability is also an issue.

##### *7.1.1 In-situ real-time decision-making*

In the traffic domain, few tools support in-situ real-time decision-making based on effective urban online monitoring and offline diagnoses, even though about half of urban visual analytics studies are concerned with traffic problems. Improving traffic flows is the most important issue in the traffic domain. When congestion is detected in real time, what diversions and other measures can be taken to alleviate the congestion and improve traffic flows, and which is best? This requires traffic monitoring and prediction, mobility analysis, and comparative analysis support in visual analytics.

##### *7.1.2 City-wide disease spread*

Affected by COVID-19, the issue of public health safety has become deeply rooted in people's hearts. Many visualization methods exist for studying the spread of disease [112, 113, 195], but few focus on infectious diseases in a fine-grained way in urban space. Health departments need an uncertainty-aware visual analytics system that integrates mobility patterns and disease transmission models to assess potential risks within geographic regions, as well as identifying vulnerable individuals. At the same time, privacy protection is an important issue in such public health analyses.

##### *7.1.3 Data availability*

Finally, we believe that the rapid development of urban analytics in the traffic domain can partially be attributed to the availability of public traffic datasets. We call on practitioners of urban analytics, whether in industry or academia, to release more high-quality, interesting data [3, 196, 197], and thereby promote urban analytics research in various domains.

#### 7.2 Visualizations

We now indicate two types of visualization techniques we believe are worth investigating in future.

##### *7.2.1 Integrated visualization*

We agree with previous surveys that urban data can always be classified as having spatial, temporal, and other properties [7, 9]. Many specific visualization methods have been designed for these three kinds of data, and it is non-trivial to propose something novel given these excellent visual designs. Nevertheless, it is still a promising research direction to effectively integrate spatial and temporal information [152, 173, 198, 199].

##### *7.2.2 AI4VIS*

Rich visualization datasets [200, 201] and powerful artificial intelligence (AI) models have yielded an emerging and promising research topic called AI4VIS [202, 203]. Researchers have started to leverage AI to generate or recommend visualizations given an input dataset. Existing AI4VIS methods mainly focus on tabular data and basic charts (e.g., bar charts) for information visualization, while visualization of spatiotemporal data in urban space has so far been ignored. We can imagine in future a set of effective spatiotemporal visualizations which are automatically generated and coordinated for urban visual analysis, given only simple inputs like a dataset and tasks. Realizing such a vision is challenging and requires long-term research effort.

### 7.3 Computational methods

Computational methods should be interpretable and tailored to scenarios.

*7.3.1 Interpretable computation*

Deep learning is still rarely used in urban visual analytics, although it is already in full swing in the field of artificial intelligence [204]. Obtaining deep insights into improved cities relies on a comprehensive understanding of urban data through methods such as frequent pattern mining, various indicators based on rules, and understanding spatial distributions by KDEs. However, deep learning currently mainly supports prediction, classification, and representation, which are only a few of the analysis functions required. Besides, its limited interpretability prevents its use in a user-centric system. Based on these observations, we believe that interpretable computational methods, such as these statistical and rule-based methods, will still prevail in urban visual analytics in the foreseeable future. At the same time, practitioners should make full use of the powerful capabilities of deep learning in prediction, classification, and representation.

*7.3.2 Scenario-tailored computation*

Visualization researchers have proposed many practical computational methods, some of which extend existing approaches. Urban visual analytics research aims to design human-in-the-loop solutions for improved cities rather than developing innovative computational methods. Nonetheless, a user-centric system may raise unique requirements on computational methods, such as being fast enough to support seamless interaction [69, 100] or being steerable [25, 189]. These requirements motivate visualization researchers to adapt state-of-the-art computational methods or couple them with visualization, thereby improving their practicality and effectiveness in urban visual analytics.

### 7.4 Systems

Among the four types of systems, visual analytics-assisted models have better prospects, as the other three are well established.

Many complicated models, particularly those based on deep learning, are increasingly applied in urban scenarios, such as autonomous driving, trajectory prediction [205], and urban flow analysis [2, 206, 207]. Despite the excellent results achieved, the dynamic and complex urban environment poses challenges when deploying such models in the real world [77–79]. Urban experts require them to be tested, debugged, and diagnosed in real scenarios. If users directly manipulate an advanced model, it may achieve better performance. A model steering mechanism is an effective way to involve human knowledge into machine intelligence: users and machines communicate closely through interactive visualization. The obvious advantages of model diagnosis and steering will encourage such urban visual analytics systems, especially as models become more powerful but complex.

### 7.5 Analytics environments

The development of hardware and collaboration technologies suggests future analytics environments for urban visual analytics, such as immersive, collaborative, and mobile environments.

*7.5.1 Immersive environments*

In recent years, the rapid development of virtual reality technologies has made immersive urban visual analytics possible [208–212]. Virtual reality devices can significantly empower urban analysts with a sense of presence by integrating the critical 3D context [150]. Nonetheless, immersive urban visual analytics is still in a very early stage. We have not seen an immersive tool that supports complex urban analysis tasks like those for desktop environments. There is an urgent need for researchers to explore effective spatiotemporal visualizations and visual analytics methods in an immersive environment.

*7.5.2 Collaborative environments*

A collaborative analytics environment is conducive to urban visual analysis. The investigation of urban phenomenons may involve multiple fields, and thus collaboration among experts from these fields is generally necessary. For example, air pollution can be caused by local traffic congestion or pollutants propagated from remote regions. In such cases, transportation experts and environmental experts need to cooperate to analyze the problem. How to apply existing collaborative analysis methods [213, 214] to urban visual analytics and what resulting challenges will be encountered remain open problems.

*7.5.3 Mobile devices*

Nowadays, mobile devices, such as phones and tablets, have become the most accessible analytics terminals. For example, a police officer with a tablet could regulate traffic at a crossroad, empowered to perform traffic analysis and take actions in real time, instead of relying on a remote command center. Unfortunately, no urban visual analytics method has been developed for mobile devices. One potential reason could be their limited computing capability and screen size, restricting complex data visualization. While researchers have made preliminary attempts in mobile information visualization [215–218], the design and development of urban visual analytics systems on mobile devices needs further investigation.

### 8 Conclusions

Urban visual analytics is an effective path towards smart cities. Developing an urban visual analytics system demands domain problem characterization and a combination of visualization and computation. This paper reviews research progress in urban visual analytics from the four perspectives of domain problem, visualization, computational analysis, and system, to provide visualization and urban analysis practitioners understanding of the state-of-the-art in urban visual analytics, as well as future research directions and opportunities. We provide an interactive tool for exploring the surveyed papers based on our typology at `https://urban-va-survey.github.io/`.

#### Acknowledgements

This work was supported by National Natural Science Foundation of China (62072400), the Collaborative Innovation Center of Artificial Intel-ligence by MOE and Zhejiang Provincial Government (ZJU), and the Zhejiang Lab (2021KE0AC02).

#### Declaration of competing interest

The authors have no competing interests to declare that are relevant to the content of this article.
